# Editorial: The cognitive ageing collection

**DOI:** 10.1038/s41598-024-60763-7

**Published:** 2024-05-13

**Authors:** Louise A. Brown Nicholls, Martina Amanzio, Bahar Güntekin, Hannah Keage

**Affiliations:** 1 Department of Psychological Sciences and Health, University of Strathclyde, 40 George Street, Glasgow, G1 1QE UK; 2 Department of Psychology, University of Turin, 10024 Turin, Italy; 3 Department of Biophysics, School of Medicine, and Research Institute for Health Sciences and Technologies (SABITA), Neuroscience Research Center, Istanbul Medipol University, Istanbul, Turkey; 4 Discipline Psychology, Justice and Society, University of South Australia, GPO BOX 2741, Adelaide, 5000 Australia

**Keywords:** Psychology, Neuroscience

## Abstract

Alongside rapid population ageing, we are experiencing increasing numbers of people with cognitive impairment and dementia. There is great scientific effort being committed to understanding cognitive and brain functioning, with the aim of helping to promote healthy ageing and independence, and improve quality of life. This Cognitive Ageing Collection brings together cutting-edge research using a variety of methods and from diverse disciplinary perspectives, with example topics including cognitive strategies, genetic risk factors, and emotion regulation. Articles in the Collection highlight advances in our understanding of cognitive and brain health, and outline important directions for future research.

Globally, we are experiencing rapid population ageing. The number of older adults will likely double by 2050 to 2.1 billion, with the most rapid increases occurring in developing countries^1,2^. Population ageing brings many positives for people and society, but there are associated social, healthcare, and economic challenges. For example, the number of people living with dementia will likely increase from 55 million in 2019 to 139 million by 2050^3^. During the United Nations Decade of Healthy Ageing 2021–2030^2^, concentrated and sustained action is required to understand and promote healthy ageing, and to help prevent or delay impairment. This Cognitive Ageing Collection brings together cutting-edge research in cognition and brain health, contributing to the advancement of scientific understanding in this important and timely topic.

Some cognitive abilities, especially those based on knowledge and prior experience, such as verbal knowledge or wisdom, tend to remain stable or continue improving through the adult lifespan^4,5^. Others, especially those involving speeded information processing or attentional control, such as working memory and executive function, exhibit gradual decline from the third decade of life^6,7^. In this Collection, Nyberg et al.^8^ highlight the heterogeneity in five-year cognitive ageing trajectories between individuals (see also Ref.^9^). They show that stability versus decline of working memory performance is related to ‘brain maintenance’, using both task-related (e.g., prefrontal cortex activity) and broader (e.g., hippocampal and ventricular volume) neural integrity measures.

There is great potential for the brain to compensate for age-related functional and structural brain changes. ‘Cognitive reserve’, accumulated over the lifespan, for example via education and other stimulating activities, may act as a buffer against cognitive impairment^10,11^. Cognitive reserve is associated with improved performance in global cognition, executive functions and attention^12,13^ and has been shown to protect against brain damage and dementia, slow the cognitive aging process, and reduce the risk of psychiatric disorders^14,15^.

Interestingly, a longitudinal neuropsychophysiological study published as part of this Collection examined University of the Third Age participants who continued to attend their learning classes and share their experiences via social media during the COVID-19 pandemic^16^. Neuropsychological data from before and during the pandemic showed that older people were able to maintain their cognitive ability if they continued to participate in social and educational activities. Cognitive reserve not only helped people to cope with negative experiences, but also played a protective role in emotional dysregulation during the pandemic. This was determined by recording heart rate variability while participants were shown images reminiscent of the most dramatic lockdown phase. Thus, preserved global cognition played a protective role against the effects of pandemic-induced anxiety and emotional dysregulation on apathy.

Multiple evidence-based approaches are being tested as potential interventions for optimizing cognitive and brain health (e.g., cognitive, physical, and/or social engagement^17^). One approach to compensation is to draw upon acquired knowledge, which typically increases through the lifespan, to help support aspects of functioning that are more vulnerable to ageing^18^. Recent literature has also been addressing the specific strategies individuals can adopt during cognitive tasks. For example, to what extent might older adults use less efficient or varied strategies than young adults^19,20^? In the current Collection, Radnan^21^ show that memory compensation is used across the adult lifespan to support everyday cognitive tasks such as remembering an appointment. In both young (17–25 years) and older (60–89 years) adults, the use of ‘external’ strategies (e.g., writing a list) was more prevalent than internal strategies (e.g., using a mnemonic). However, older adults reported using more strategies overall, and using more physical and environmental tools. They generally exhibited high uptake of digital tools, but they were still less likely than younger adults to report using either digital or social tools. More research is needed to understand cognitive strategy use across the adult lifespan, and the potential for strategy training to boost older adults’ performance^22,23^.

Resilience to cognitive decline and dementia, such as Alzheimer’s disease, may operate differently for men and women. In this Collection, Zheng et al.^24^ identified factors that were associated with resilience to cognitive decline in APOE ɛ4 carriers (the largest genetic risk factor for late-life dementia). These were increased mild physical activity and being employed for men, and increased mental engagement for women. When modelling dementia as an outcome, we must also consider mortality. In this Collection, Pirraglia et al.^25^ demonstrated that APOE ɛ4 was associated with decreased mortality risk in people who died with a low burden of Alzheimer’s neuropathology, but APOE ɛ4 was associated with increased mortality risk in people who died with a high burden of Alzheimer neuropathology.

Emotional stimulation, perception, and regulation can be highly affected during cognitive aging. Older adults are biased towards positive stimuli (i.e., the positivity effect^26–28^). They also rate positive stimuli as less arousing, and negative stimuli as more arousing, than younger adults^29–32^. This finding was confirmed by Lee et al.^33^ in the present Collection. Emotions affect memory processes such that high-arousal stimuli are remembered better than low-arousal stimuli. So, the dysregulation of emotion affects not only older adults’ emotional states, but also their memory performance. Lee et al.^33^ showed that memory enhancement linked to arousal appears to diminish with age, particularly in the context of negative stimuli.

Recognising facial expressions can also change with age^34^. In this Collection, Horta et al.^35^ explored how age groups differ in their responses to facial trustworthiness, investigating its influence on decision-making and learning. They showed that both young and older age groups preferred trustworthy faces. However, across all tasks, older adults consistently underperformed over time, compared to their younger counterparts.

Another neuropsychophysiological study included in the Collection analyzed the response of healthy older adults during the COVID-19 pandemic. That is, the emotional impact of the pandemic approximately 20 months after the outbreak was investigated using a unique interdisciplinary approach^36^. Emotional (dys)regulation was studied using facial electromyographic recordings during the presentation of COVID-19-related images. Such stressful stimuli triggered the activation of bottom-up emotion strategies associated with higher mood levels and interacted with top-down factors that play an important role in the dysregulation of cognitive control.

This Collection showcases key findings and issues in the study of cognitive ageing. We have highlighted a selection of articles from the Collection that illustrate the extensive research methods and topic areas that are covered. The field has clearly been making great advances, on which we must continue to build. For example, future research requires more diverse samples to be recruited, so that we can understand the trajectories of populations rather than selected samples. Furthermore, we need to increase understanding across the range of cognitive domains, including those less studied, such as social cognition.
